# Transcriptome and hormone profiling reveals *Eucalyptus grandis* defence responses against *Chrysoporthe austroafricana*

**DOI:** 10.1186/s12864-015-1529-x

**Published:** 2015-04-18

**Authors:** Ronishree Mangwanda, Alexander A Myburg, Sanushka Naidoo

**Affiliations:** Department of Genetics, Forestry and Agricultural Biotechnology Institute (FABI), Genomics Research Institute (GRI), University of Pretoria, Private bag x20, Pretoria, 0028 South Africa

**Keywords:** *Eucalyptus* stem canker, Gibberellic acid, Hormone signalling, Plant defence, RNA-sequencing, Salicylic acid

## Abstract

**Background:**

*Eucalyptus* species and interspecific hybrids exhibit valuable growth and wood properties that make them a highly desirable commodity. However, these trees are challenged by a wide array of biotic stresses during their lifetimes. The *Eucalyptus grandis* reference genome sequence provides a resource to study pest and pathogen defence mechanisms in long-lived woody plants. *E. grandis* trees are generally susceptible to *Chrysoporthe austroafricana*, a causal agent of stem cankers on eucalypts. The aim of this study was to characterize the defence response of *E. grandis* against *C. austroafricana*.

**Results:**

Hormone profiling of susceptible and moderately resistant clonal *E. grandis* genotypes indicated a reduction in salicylic acid and gibberellic acid levels at 3 days post inoculation. We hypothesized that these signaling pathways may facilitate resistance. To further investigate other defence mechanisms at this time point, transcriptome profiling was performed. This revealed that cell wall modifications and response to oxidative stress form part of the defence responses common to both genotypes, whilst changes in the hormone signaling pathways may contribute to resistance. Additionally the expression of selected candidate defence response genes was induced earlier in moderately resistant trees than in susceptible trees, supporting the hypothesis that a delayed defence response may occur in the susceptible interaction.

**Conclusion:**

The ability of a host to fine-tune its defence responses is crucial and the responses identified in this study extends our understanding of plant defence, gained from model systems, to woody perennials.

**Electronic supplementary material:**

The online version of this article (doi:10.1186/s12864-015-1529-x) contains supplementary material, which is available to authorized users.

## Background

*Eucalyptus,* a member of the myrtle family, is a genus of woody plants that are keystone ecological species in their natural range in Australia and nearby islands. Eucalypt species and hybrids are a valuable international commodity due to their superior growth and wood properties benefiting timber, pulp and paper production. These trees are also being investigated as a potential lignocellulosic feedstock for biofuel and biomaterials production [1,2]. Long-lived plants such as eucalypts encounter various abiotic and biotic stresses throughout their lifetimes that affect growth and the quality of the wood at rotation age. An investment in maintaining healthy trees is thus important for ensuring future sustainability of the forestry industry [3]. Current disease control strategies such as hygiene practices in nurseries are short-term solutions thus other avenues should be explored to further understand how eucalypts respond during biotic stresses.

The availability of the *Eucalyptus* genome provides an invaluable reservoir to mine for information on various responses such as those activated following an encounter with a pathogen [4,5]. Plant defences have been extensively studied in model organisms such as *Arabidopsis thaliana* and *Nicotiana spp.* but information about this is limited in eucalypts [6]. From these model systems, it has emerged that plant defence is a multi-faceted and complex process that requires fine-tuning by the host. Perception of a pathogen occurs through receptors in the cell membrane that transduce the signal through various signaling cascades [7,8]. This transduction results in the initiation of a plethora of mechanisms that alter pathogen proliferation such as the generation of reactive oxygen species (ROS), cell wall modifications, hormone signaling and the expression of defence-related proteins [9-11]. The host needs to be able to tightly regulate these responses as defence is a costly endeavor and these adaptations are usually dependent on the lifestyle of the pathogen. Biotrophic pathogens are restrained through the production of ROS and an induction of the salicylic acid (SA) pathway. However, necrotrophic pathogens can thrive on dead tissue and the production of ROS creates a favorable environment for the pathogen which may further promote its proliferation. Defence against necrotrophs then usually involves triggering the ethylene (ET) and jasmonic acid (JA) pathways [9].

*Chrysoporthe austroafricana* is considered a fungal necrotroph that causes the development of stem cankers on *E. grandis* thereby reducing wood quality, viability and growth [12,13]. Although a devastating pathogen in the late 1990’s, the spread of this fungal pathogen is currently controlled through the vegetative propagation of *E. grandis x E. urophylla* hybrids. This interaction between *E. grandis* and *C. austroafricana*, can be used as a model and one can exploit this system to expand our understanding of defence in woody species by investigating the responses of a moderately resistant and susceptible host. In the interaction with *C. austroafricana*, the *E. grandis* clone TAG5 is moderately resistant whilst *E. grandis* ZG14 is susceptible, with lesion lengths twice that of TAG5 [14]. With the availability of the *E. grandis* genome sequence, this type of study can provide insight into the defence mechanisms employed by the host. Thus the aim of this study was to identify putative defence responses triggered in *E. grandis*, following challenge by the fungal pathogen *C. austroafricana*. This was achieved by profiling known phytohormones and identifying the transcriptional responses activated in the moderately resistant and susceptible hosts through RNA-sequencing. The power of comparing these interactions lies in the ability to deduce basal and induced defence mechanisms that contribute to limiting the spread of the pathogen but also exposes weakness of the host that the pathogen may manipulate. We find that although there was an overlap between the two hosts in terms of general defence, the moderately resistant host was able to regulate defence responses such as hormone levels that may limit the spread of the disease.

## Results

### Disease progression of *C. austroafricana* on TAG5 and ZG14

**Figure 1:**
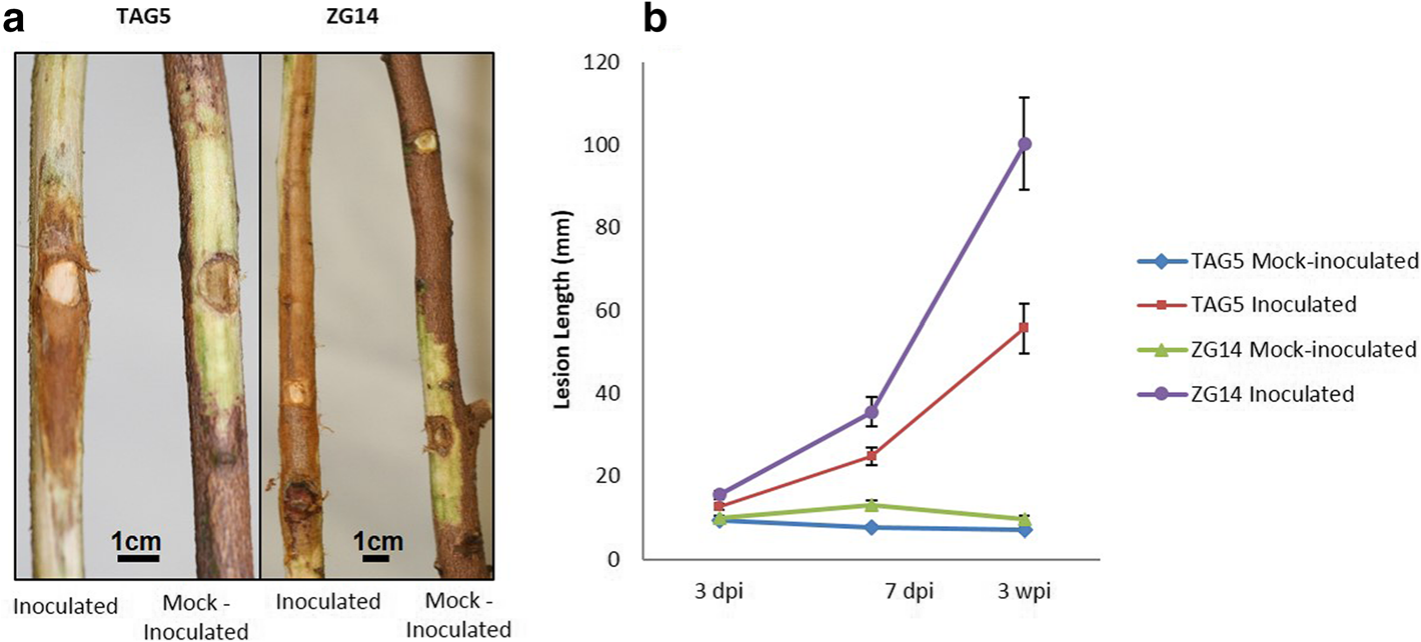
*Eucalyptus grandis* clones challenged with *Chrysoporthe austroafricana*. **a** – Lesion development in TAG5 and ZG14 at 3 weeks post inoculation. **b** – Progression of lesion lengths over a 3 week period in TAG5 and ZG14.

Stems of *E. grandis* ramets of TAG5 and ZG14 were inoculated with *C. austroafricana* and the development of lesions was monitored over the course of six weeks. Measurements taken at 7 days post inoculation (dpi) and 3 weeks post inoculation (wpi) showed a clear difference in lesion development between TAG5 and ZG14 (Figure 1). The reduced lesion development in TAG5 was therefore consistent with the classification of this host as moderately resistant according to Van Heerden et al. [14].

No lesion development was observed in the mock-inoculated ramets. While there is indication that wounding would have an effect on a host’s defence responses as observed in model systems [15], the current experiment was designed to use a wounded mock inoculated control. This type of inoculation closely reflects a natural mode of entry of the fungus in *Eucalyptus* plantations. While an unwounded control would provide insight into the wound response due to the inoculation procedure, we considered the mock-inoculated control as a more biologically relevant comparison to study the effects of the fungus directly.

### Hormone profiling highlights changes occurring at 3 dpi in TAG5 that may contribute to resistance

Phytohormones form a critical facet of the defence cascade and therefore it was of interest to investigate how these metabolites are influenced in *E. grandis* under biotic stress. Thus to further evaluate the role of these phytohormone in the interaction with *C. austroafricana*, we measured JA, SA and gibberellic acid (GA) at various time points in TAG5 and ZG14. This hormone profiling revealed patterns in TAG5 and ZG14 that may contribute to resistance or susceptibility respectively.

**Figure 2:**
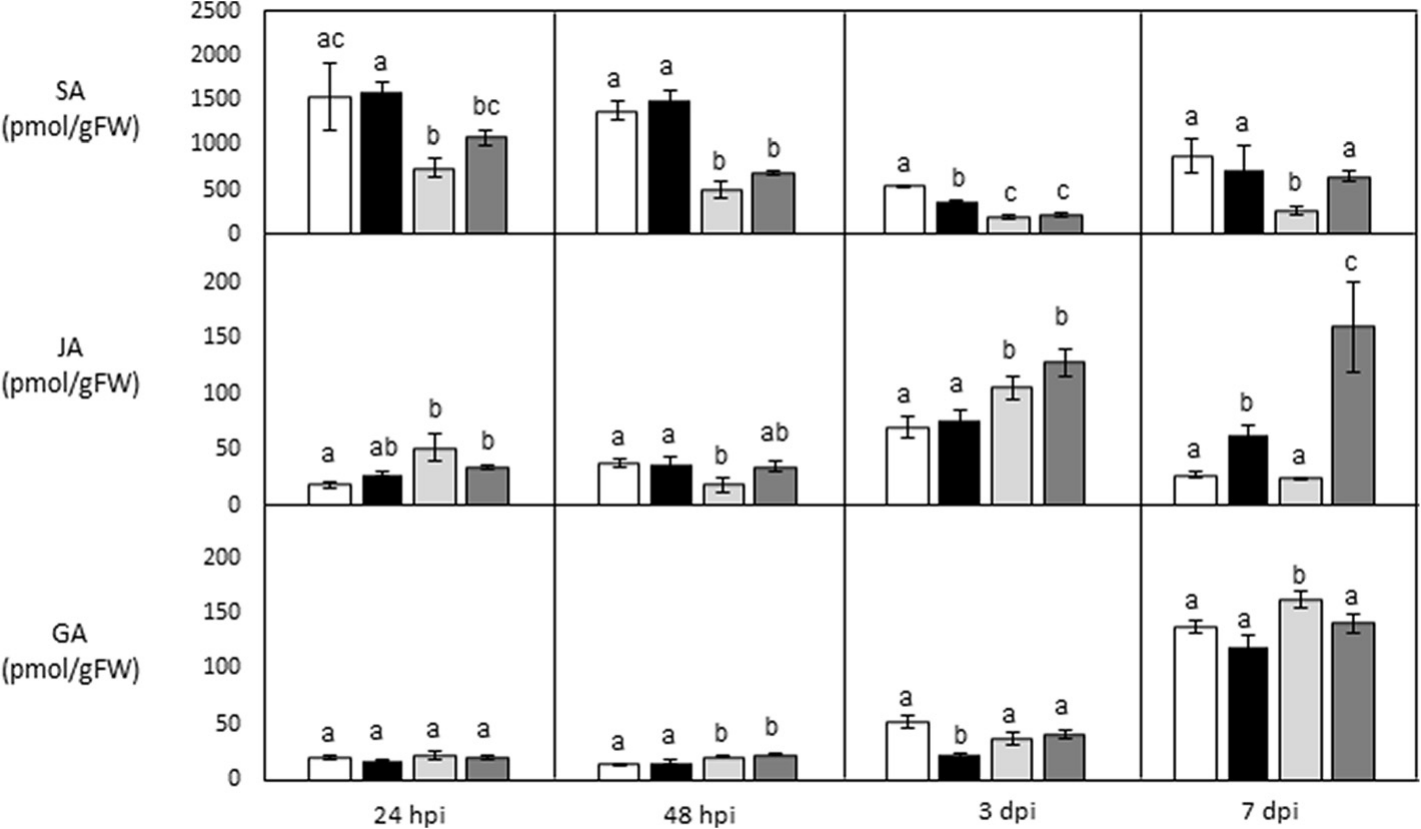
Hormone measurements of TAG5 and ZG14 over a time series post challenge with *C. austroafricana*. TAG5 mock-inoculated (White bars) and TAG5 inoculated (black bars). ZG14 mock-inoculated (Light grey bars) and ZG14 inoculated (Dark grey bars). Error bars are indicative of the standard error of the mean of the biological replicates (n = 3) for each sample. Significant differences are denoted by different alphabets (Kruskal-Walis test, p < 0.05).

At the earlier time points (24 hours post inoculation (hpi) & 48 hpi), no significant changes were observed in TAG5 or ZG14 with the hormones profiled (Figure 2). SA was found to occur at significantly higher basal levels in the moderately resistant host TAG5, than in the susceptible ZG14 at all the time points however, following inoculation, SA decreases in TAG5 at 3 dpi but remains constant in ZG14 (Figure 2). Interestingly at 3 dpi, a decrease was also observed for GA in TAG5 after pathogen challenge but no change was found to occur in ZG14. At 7 dpi, an increase of JA was observed in TAG5 and ZG14, which also displayed changes in SA and GA having increased and decreased respectively. Concurring with antagonism between SA and JA, the high basal levels of SA in TAG5 correlated with a lower basal level of JA in TAG5 than in ZG14 (Figure 2).

This data alludes to significant changes occurring with the phytohormones at 3 dpi in TAG 5 that are absent in ZG14 and may contribute to resistance. Additionally there was no significant differences observed in the lesion lengths between the genotypes at 3 dpi (Figure 1) and therefore this time point was selected to further investigate additional transcriptional changes that may elucidate other defence mechanisms.

### Transcriptome profiling of TAG5 and ZG14 challenged with *C. austroafricana*

**Figure 3:**
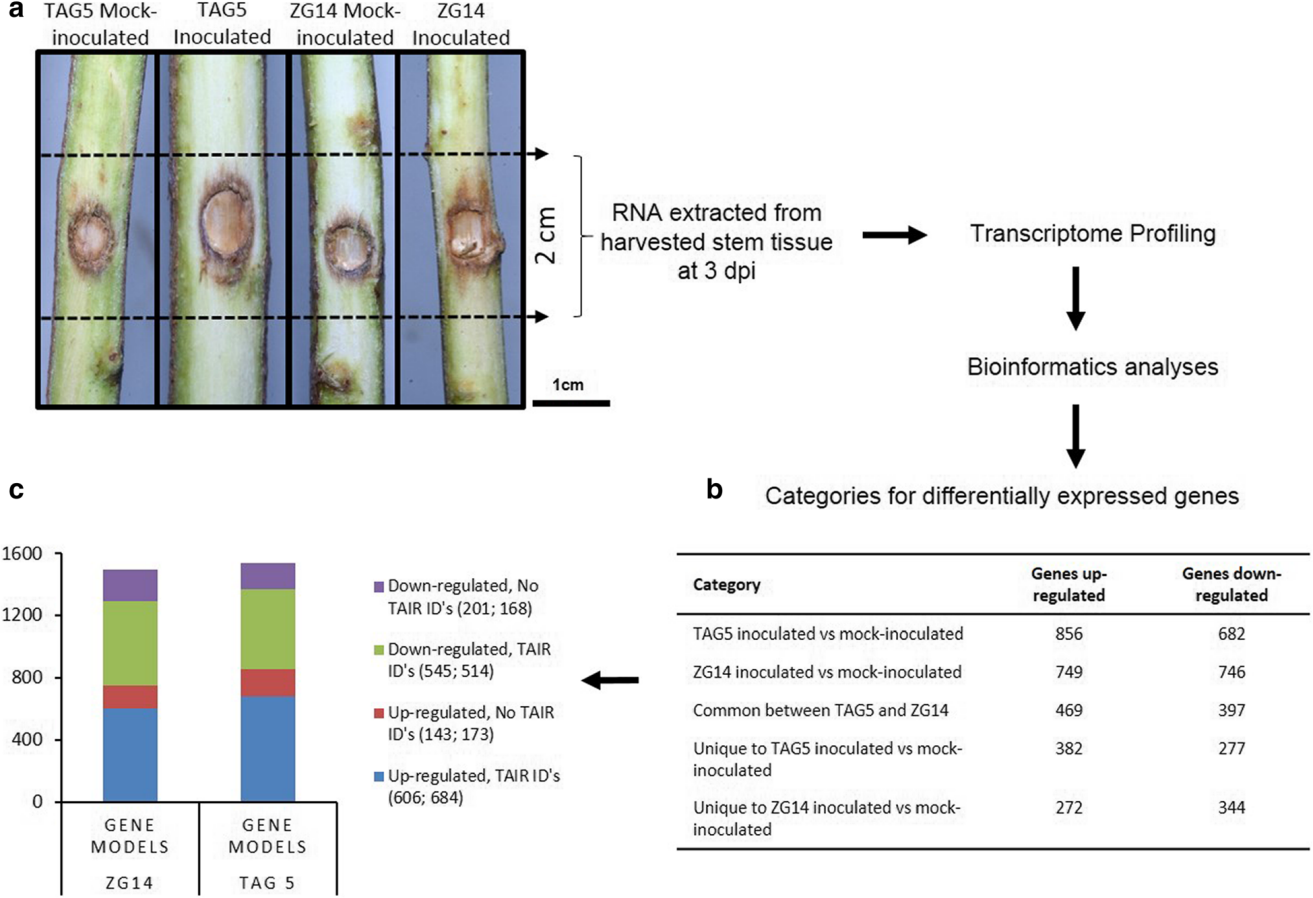
Schematic representation of transcriptome profiling conducted with *E. grandis* challenged with *C. austroafricana*. **a** - Lesion development at 3 dpi. **b** – Categories of significantly differentially expressed genes indicating the degree of uniqueness and similarity in the datasets. **c** - Bar graph representing the number of *E. grandis* gene models that had corresponding *A. thaliana *ID’s.

Transcriptome profiling of TAG5 and ZG14 at 3 dpi (Figure 3) yielded at least 34 million total paired ends reads in each sample which all passed quality control assessment (Additional file 1: Table S1). Over 75% of the reads were found to map to the *E. grandis* genome. Additionally, 2-3% of the reads from the infected samples mapped to the genome of the pathogen, *C. austroafricana* (Additional file 1: Table S1). The *E. grandis* genome currently has 36,376 predicted protein coding genes [4]. Cufflinks analysis of our datasets identified between 27,714 and 29,829 expressed genes (FPKM > 0) (Additional file 1: Table S1). Cuffdiff analyses identified 1539 and 1495 significantly DE gene models (inoculated vs controls) in TAG5 and ZG14 respectively (Figure 3b). The full list of expressed genes can be found in Additional file 2: Table S2. These gene lists were used for further analyses to elucidate patterns of defence responses emerging from the datasets.

### Over-representation of GO terms within the datasets reveals distinct defence responses

To further investigate patterns of the activated defence responses within each host, DE genes that had corresponding *Arabidopsis* identities (Figure 3c) were analyzed to identify over-representation of gene ontology terms within the three categories: molecular function (MF), cellular component (CC) and biological processes (BP). This analysis provided an indication of the overall changes occurring within each GO category and allows for a broad comparison of the processes occurring in TAG5 and ZG14 at the different stages of plant defence. It also enables the detection of differences in the patterns observed between a resistant and susceptible host indicated by the presence or absence of GO terms. Only terms that had a log_2_ (q-value) > 10 are indicated on the graph and this was applied for all GO categories except the biological processes.

 For both the up-regulated and down-regulated datasets, the MF and CC categories highlighted the presence of over-represented GO terms that were either shared between host responses or unique to a genotype. For the up-regulated dataset in the MF category, oxidoreductase activity was present in both datasets as genes within this term are generally associated with controlling ROS during a defence response (Additional file 3: Figure S1). Other terms such as “protein transmembrane transporter activity” was found only in TAG5. In the MF down-regulated dataset, there exists a clear difference in “transcription regulator activity” and “chlorophyll binding” between the two hosts. Interestingly, for the CC category, there were more terms unique to the moderately resistant TAG5 in the up-regulated dataset, while there were more terms unique to the susceptible ZG14 in the down-regulated dataset (Additional file 4: Figure S2). Amongst the shared terms of the up-regulated datasets for CC was the “cell wall” category, which had more over-represented terms in TAG5 than in ZG14.

**Figure 4:**
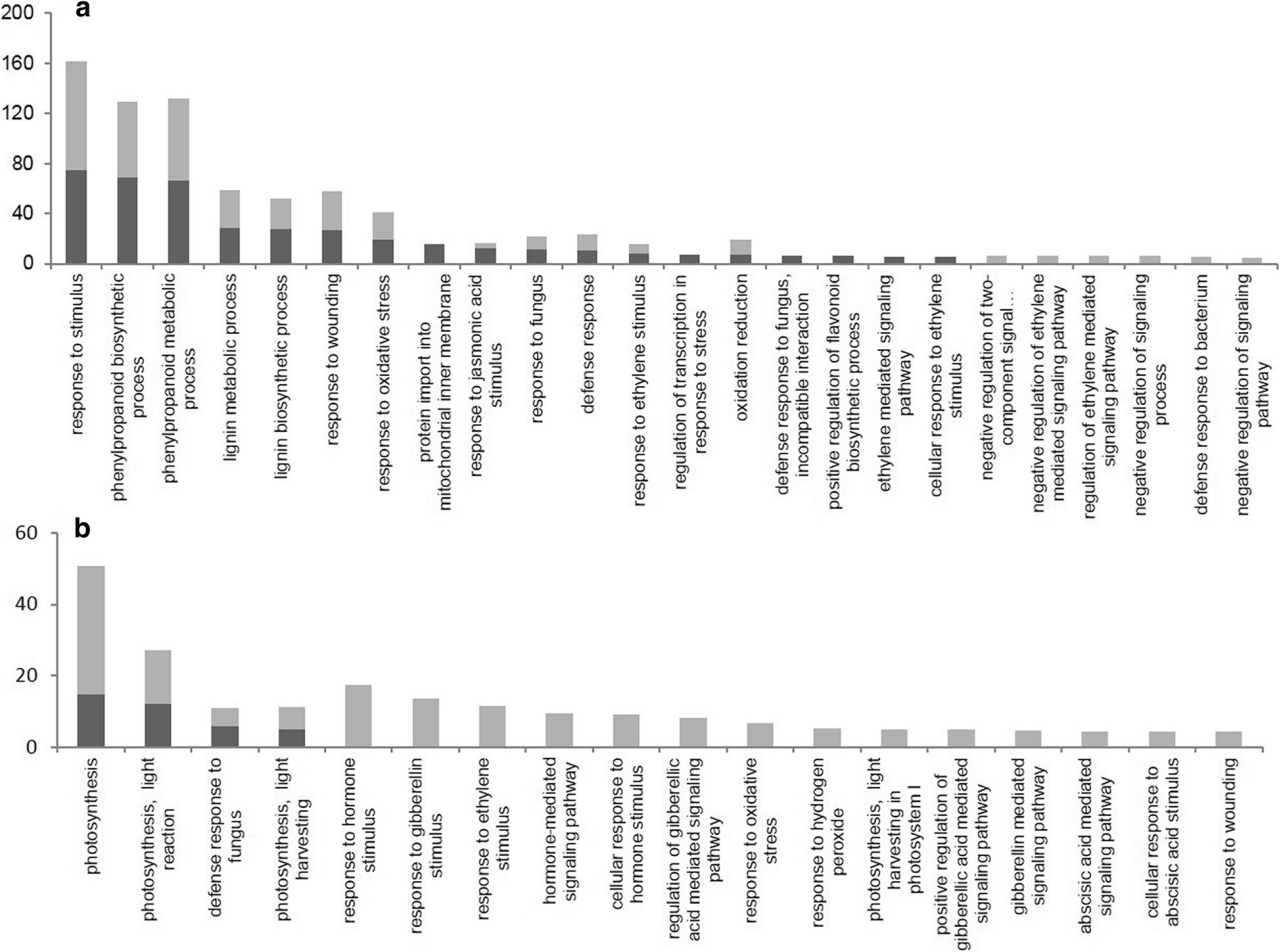
Selected biological process GO terms that are over-represented in TAG5 and ZG14. **a** – BinGO terms within the up-regulated dataset. **b** – BinGO terms within the down-regulated dataset for TAG5 (Dark grey bars) and ZG14 (Light grey bars). The y-axis represents the –log_2_(q-value) obtained with the Benjamini & Hochberg False Discovery Rate (FDR) correction analysis. The x-axis represents the BinGO terms within the datasets.

Due to the large array of BP responses that were observed, only key processes are highlighted in the figures. These processes were selected based on known categories of defence that are affected during pathogen interactions. Both hosts contained terms in the up-regulated datasets associated with the phenylpropanoid pathway, response to oxidative stress, secondary metabolic process, lignin metabolic processes, response to ethylene and response to jasmonic acid. In the ZG14 up-regulated dataset, there was an over-representation of terms involved in the negative regulation of the ET pathway such as “negative regulation of ethylene-mediated signaling pathway” (Figure 4a). This latter term encompasses genes such as *EBF1/2* which is involved in suppressing ET signaling. In TAG5, the term “defence to fungus, incompatible interaction” was found but did not appear within the ZG14 dataset. This corroborates the hypothesis that TAG5 is moderately resistant and is able to withstand pathogen infection to a degree (Figure 4a).

Within the down-regulated dataset, there was an over-representation of photosynthesis-related terms in both TAG5 and ZG14. However, ZG14 also has over-represented terms such as “abscisic acid mediated signaling pathway” and “response of gibberellic acid mediated signaling” that are absent from the TAG5 dataset (Figure 4b). The candidates within the BP GO term categories were subsequently further analyzed to highlight any key distinctions in different metabolic processes.

### Differences in the regulation of defence responses occur in the two hosts

Differentially expressed (DE) gene models from TAG5 and ZG14 up – and down-regulated datasets were analyzed with MAPMAN which provides a visual representation of the genes within different cellular processes. Due to the large degree of overlap between the two hosts, the DE gene lists were subdivided into sets of genes DE in TAG5 and ZG14, those unique to TAG5 (659 gene models, Figure 3) and those unique to ZG14 (616 gene models, Figure 3).

#### Defence responses shared between TAG5 and ZG14

From the 866 gene models common to both hosts (Figure 3b), 809 gene models were found to have corresponding *A. thaliana* IDs. The gene models that were found to be common amongst the resistant and susceptible interactions correlated with many processes that are affiliated with a defence response. These processes included up-regulation of genes involved in hormone signaling such as JA and ET, an increase in the number of secondary metabolites, changes in the cell wall structure, up-regulation of pathogenesis related proteins as well as the respiratory burst. Although the terms may have been shared between the hosts, the expression levels of the genes associated with these terms were found to be expressed at different levels. Each of these categories also contained genes that were both up- and down-regulated indicating a tight regulation between cellular processes (Table 1; Table 2).

**Table 1:** **Genes involved in phytohormone signalling with differential expression (log**_**2**_ **) in TAG5 and ZG14 following challenge with** ***C. austroafricana***

**TAIR ID**	**Euc ID**	**Description**	**Abbreviation**	**TAG 5**	**ZG14**
**SA**					
AT2G38470.1	Eucgr.B04010	WRKY DNA-binding protein 33	WRKY33	1.00	1.50
AT2G23620.1	Eucgr.I01005	Methyl esterase 1	ATMES1	3.69	N/A
AT2G18660.1	Eucgr.C00204	Plant natriuretic peptide A	PNP-A	0.87	−0.63
**JA/ET**					
AT3G23240.1	Eucgr.K03266	Ethylene response factor 1	ERF1, ATERF1	2.97	1.53
AT5G47220.1	Eucgr.E02651	Ethylene responsive element binding factor 2	ATERF2	2.86	N/A
AT2G25490.1	Eucgr.C01524	EIN3-binding F box protein 1	EBF1, FBL6	N/A	1.11
AT5G25350.1	Eucgr.C01334	EIN3-binding F box protein 2	EBF2	N/A	1.13
AT3G16770.1	Eucgr.H03965	Ethylene-responsive element binding protein	RAP2.3, ERF72	1.24	N/A
AT2G19590.1	Eucgr.C03886	ACC oxidase 1	ACO1	3.59	N/A
AT1G05010.1	Eucgr.K00739	Ethylene-forming enzyme	EFE, ACO4	3.15	2.26
	Eucgr.K00749	Ethylene-forming enzyme	EFE, ACO4	1.99	N/A
	Eucgr.K00747	Ethylene-forming enzyme	EFE, ACO4	1.91	N/A
AT5G42650.1	Eucgr.F01505	Allene oxide synthase	AOS, CYP74A	0.81	0.88
AT1G55020.1	Eucgr.H04496	Lipoxygenase 1	LOX1, ATLOX1	−1.12	−1.68
AT3G22400.1	Eucgr.H03535	Lipoxygenase family protein 5	LOX5	−1.75	−1.75
AT3G12500.1	Eucgr.I01495	Basic chitinase	ATHCHIB, PR3	1.94	1.39
	Eucgr.L00937	Basic chitinase	ATHCHIB, PR3	1.66	1.60
	Eucgr.J02519	Basic chitinase	ATHCHIB, PR3	1.35	1.76
	Eucgr.J02518	Basic chitinase	ATHCHIB, PR3	0.85	N/A
AT3G04720.1	Eucgr.B02122	Pathogenesis-related 4	PR4, HEL	1.26	N/A
	Eucgr.H04329	Pathogenesis-related 4	PR4, HEL	N/A	−1.49
AT2G14580.1	Eucgr.G01134	Basic pathogenesis-related protein 1	ATPRB1, PRB1	1.25	N/A
AT4G11650.1	Eucgr.D01888	Osmotin 34	ATOSM34	0.93	0.93
AT1G19180.1	Eucgr.C03301	Jasmonate-zim-domain protein 1	JAZ1	1.95	2.05
**GA**					
AT1G30040.1	Eucgr.F03208	Gibberellin 2-oxidase	GA2OX2	2.75	2.19
AT1G75750.2	Eucgr.F00588	GAST1 protein homolog 1	GASA1	−1.13	−0.90
	Eucgr.F00590	GAST1 protein homolog 1	GASA1	N/A	−0.85
AT3G02885.1	Eucgr.F02851	GAST1 protein homolog 5	GASA5	N/A	0.77
AT2G01570.1	Eucgr.G02163	DELLA protein	RGA	N/A	−0.67
AT3G03450.1	Eucgr.J01594	DELLA protein	RGL2	N/A	−0.66
AT4G17230.1	Eucgr.E03895	SCARECROW-like 13	SCL13	−0.64	1.34

Absence of candidate expression is indicated by N/A.

**Table 2:** **Genes involved in perception and early signal transduction with a differential expression (log**_**2**_ **) in TAG5 and ZG14 following challenge with** ***C. austroafricana***

**TAIR ID**	**Euc ID**	**Description**	**Abbreviation**	**TAG 5**	**ZG14**
***Cell wall***					
AT1G48100.1	Eucgr.H04092	Pectin lyase-like superfamily protein		3.33	N/A
AT1G60590.1	Eucgr.B03312	Pectin lyase-like superfamily protein		2.74	N/A
AT3G61490.3	Eucgr.E00105	Pectin lyase-like superfamily protein		2.67	1.25
AT5G04310.1	Eucgr.B03017	Pectin lyase-like superfamily protein		0.88	N/A
AT2G45220.1	Eucgr.E01463	Pectin methylesterase inhibitor	PMEI	6.18	2.57
AT4G32410.1	Eucgr.C01769	Cellulose synthase 1	CESA1	−0.95	−0.74
AT5G44030.1	Eucgr.A01324	Cellulose synthase 4	CESA4/ IRX5	−2.79	N/A
***ROS***					
AT5G47910.1	Eucgr.J01662	Respiratory burst oxidase homologue D	ATRBOHD	2.16	1.74
AT1G64060.1	Eucgr.E00785	Respiratory burst oxidase protein F	ATRBOHF	1.75	1.24
AT2G31570.1	Eucgr.A00055	Glutathione peroxidase 2	ATGPX2, GPX2	0.72	N/A
AT1G71695.1	Eucgr.F04198	Peroxidase superfamily protein		2.85	1.76
AT2G37940.1	Eucgr.K03013	Arabidopsis Inositol phosphorylceramide synthase 2	AtIPCS2	0.82	N/A
***Signalling***					
AT1G21270.1	Eucgr.F01829	Wall-associated kinase 2	WAK2	−1.45	N/A
AT4G23650.1	Eucgr.E00806	Calcium-dependent protein kinase 6	CDPK6, CPK3	0.79	N/A
AT5G23950.1	Eucgr.A02756	Calcium-dependent lipid-binding (CaLB domain)		−0.83	N/A
AT1G18210.2	Eucgr.F00233	Calcium-binding EF-hand family protein		−1.34	−1.89
AT1G70810.1	Eucgr.G02165	Calcium-dependent lipid-binding (CaLB domain)		−1.54	−1.30
AT1G24620.1	Eucgr.F04374	EF hand calcium-binding protein family		N/A	−1.52
AT4G13440.1	Eucgr.H02910	Calcium-binding EF-hand family protein		N/A	−3.85
AT4G33000.2	Eucgr.F03125	Calcineurin B-like protein 10	CBL10	−1.13	−0.66
AT1G66400.1	Eucgr.F02840	Calmodulin like 23	CML23	N/A	1.10
AT5G42380.1	Eucgr.F03632	Calmodulin like 37	CML37	N/A	1.10
AT3G45640.1	Eucgr.J00966	Mitogen-activated protein kinase 3	MPK3	N/A	1.01
AT1G73500.1	Eucgr.H00554	MAP kinase kinase 9	ATMKK9,MKK9	N/A	−0.86

Absence of candidates expression is indicated by N/A.

Hormone pathways, ET and JA, have been extensively shown to be involved in resistance against necrotrophs and thus it was postulated that these pathways would be induced in *Eucalyptus* (Table 1). In accordance, genes associated with ET that were found to be up-regulated in both hosts include: *ETR2, ACO4* and *ERF1*. In terms of DE JA genes, *LOX1* and *LOX5* were found to be down-regulated whereas *AOS* and *JAZ1* were found to be up-regulated in TAG5 and ZG14. In addition to hormone signaling, both hosts were found to have a significant number of genes involved in the phenylpropanoid pathway thus potentially implicating this pathway in defence against *C. austroafricana*. These included candidates such as phenylalanine ammonia-lyase (PAL), O-methyltransferase (OMT), caffeoyl CoA and cinnamyl-alcohol dehydrogenase.

To facilitate the large energy requirement of a defence response, the host could potentially shunt resources from other metabolic processes such as photosynthesis. Evidence of this diversion was found in the down-regulated datasets of both hosts which highlighted photosystem I and II GO terms (Figure 4b). Cell wall degrading enzymes form part of the virulence strategy of pathogens and one of the mechanisms a host employs to resist this is through the methyl esterification of pectins by pectin methylesterase inhibitors (PMEI) [16]. Both TAG5 and ZG14 exhibited a significant up-regulation of PMEI candidates (Table 2). Damage associated molecular pattern’s (DAMP’s) formed during the degradation of the cell wall results in the activation of the signaling cascade. In plants, the primary activation of ROS during a pathogen infection occurs through NADPH oxidases, also known as Respiratory burst oxidase homologs (RboH), [17,18]. Interestingly *AtRbohF* and *AtRbohD*, both associated with defence, were found to be significantly up-regulated in TAG5 and ZG14 at 3 pi (Table 2). The signaling category revealed that genes involved in calcium signaling was up-regulated in both hosts, but the specific genes encoding for the receptors were different. Of the latter sensors, only Ca^2+^ dependent protein kinases (*CPK3/CDPK6)* and calmodulin *(CaM)* were up-regulated in TAG5 and ZG14 respectively (Table 2). The signal is then transduced through various phosphorylation events to influence transcription factors (TF) and hormone signaling.

In addition to Ca^2+^ signaling, *MPK3* was found to be significantly up-regulated in ZG14. Although *MPK3* was not DE in the moderately resistant TAG5, the expression level in terms of FPKM values of the gene was comparable between both genotypes following pathogen challenge. MPK3 is known to phosphorylate WRKY33 and the gene encoding for this TF was found to be significantly up-regulated in both hosts which may mirror the induction of *MPK3*.

#### Regulation of defence processes by TAG5 may contribute to moderate resistance

Despite a degree of overlap in the defence responses that ensue challenge with *C. austroafricana*, there are marked differences that may influence the outcome of the interaction. From the 659 and 616 gene models unique to TAG5 and ZG14 respectively, 631 and 569 gene models had corresponding *A. thaliana* identities in the respective hosts. The redox state GO category highlighted candidates that were DE only in TAG5 and these include glutathione peroxidase 2 (*GPX2*) and inositol phosphorylceramide synthase 2 (*IPCS2*) (Table 2). Within the ZG14 dataset, the genes categorized under ET were different to those expressed within the TAG5 dataset. The MAPMAN data confirmed the GO ontology results in terms of ET whereby, EIN3 BINDING F-BOX1 (EBF1) and EBF2 involved in ET suppression were found to be unique to ZG14 and not found in TAG5 (Table 1). Another candidate involved in the ET biosynthetic pathway, *ACO1*, was found to occur only in the dataset unique to TAG5 and not ZG14. ACO1 was only DE in TAG5 and the expression of this gene was significantly lower and remained unchanged in ZG14.

GA is a phytohormone that is well-known for its role in plant development but has also been shown to be involved in pathogen defence. *GASA1*, a gene associated with GA signaling was found to be significantly suppressed following infection with *C. austroafricana*, in both TAG5 and ZG14. This suppression could be due to the increase of GA 2-oxidases (*GA2ox*) in both hosts which converts active GA to an inactive form [19]. A gene known as Scarecrow-like 13 (*SCL13*), involved in GA regulation is up-regulated in the susceptible ZG14 but down-regulated in the moderately resistant TAG5 (Table 1). This highlights a time in the defence series at which GA may facilitate resistance in TAG5 and is supported by a decrease of the hormone at the metabolite level at 3 dpi. The BP GO terms highlights the “response to gibberellin stimulus” in the down-regulated dataset of the susceptible interaction. Genes within this GO term are involved in the negative regulation of GA signaling such as the DELLA genes RGA1 and RGL2 (Table 1). This down-regulation of the DELLA genes in the susceptible host could contribute to higher GA levels than in TAG5 following inoculation as observed at the metabolite level (Figure 2).

Systemic acquired resistance (SAR) employs the SA pathway to confer resistance in distal plant tissues. Although SA responsive genes such as *PR1 *were not found to be DE at 3 dpi, genes associated with SAR were found to be distinctive in TAG5 and ZG14 (Table 1). Methyl esterase 1 (*AtMES1*) and plant natriuretric acid (*PNP-A*) are candidates associated with SAR. *AtMES1* was only up-regulated in TAG5, whereas *PNP-A* was found to be up-regulated in TAG5 but down-regulated in ZG14. Thus, TAG5 may utilize SA to induce an SAR response to limit pathogen spread. Therefore although there are cellular responses similar in the two interactions, there are differences between TAG5 and ZG14 that may ultimately confer a combinatorial effect resulting in a moderately resistant phenotype.

### RT-qPCR analysis reveals a delay in defence responses in the susceptible genotype

**Figure 5:**
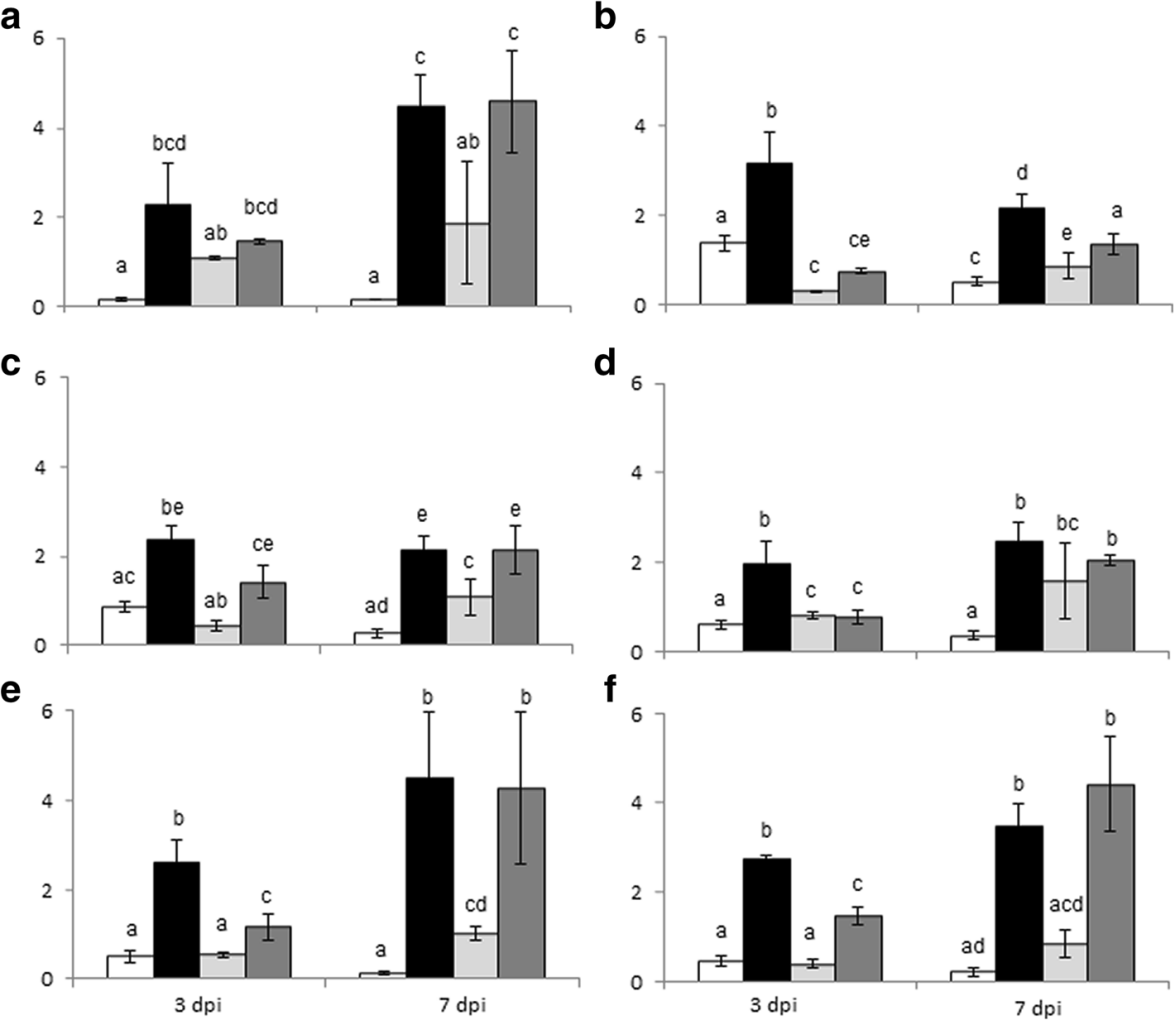
Expression profile of candidate genes from transcriptome profiling post challenge with *C. austroafricana*. **a** – *EgrACO*; **b** – *EgrIPCS*; **c** – *EgrOSM34*; **d** – *EgrPNP-A*; **e** – *EgrPR1B*; **f** - *EgrPR3*. TAG5 mock-inoculated (White bars) and TAG5 inoculated (black bars). ZG14 mock-inoculated (Light grey bars) and ZG14 inoculated (Dark grey bars). The y axis represents the relative gene expression ratios as arbitrary units. Error bars are indicative of the standard error of the mean of the biological replicates (n = 3) for each sample. Significant differences are denoted by different alphabets (Kruskal-Walis test, p < 0.05).

Candidate genes were selected for RT-qPCR profiling in order to validate the results of the transcriptome analysis. This selection was based on their transcriptome expression patterns in TAG5 and ZG14 at 3 dpi as well as their putative role in plant defence (Figure 5). The genes, 1-aminocyclopropane-1-carboxylate oxidase (*EgrACO1,* Figure 5a), inositol phosphorylceramide synthase (*EgrIPCS,* Figure 5b), plant natriuretic peptide A (*EgrPNP-A,* Figure 5d) and basic pathogenesis related 1 (*EgrPR1B,* Figure 5e) were selected as these candidates showed significant differential expression in TAG5 but not in ZG14. The other two genes, pathogenesis related protein 3 (*EgrPR3,* Figure 5f) and osmotin 34 (*EgrOSM34,* Figure 5c), are downstream defence products and that were expressed in both hosts but to a significantly higher degree in TAG5 compared to ZG14. All the candidates profiled corroborated with the RNA-seq expression patterns.

Since susceptibility might not only be due to the lack of defence activation but also due to a delayed response of the host to the pathogen, this hypothesis was investigated by profiling the above mentioned candidates at 7 dpi in TAG5 and ZG14. Interestingly for all the candidates profiled (Figure 5), except *EgrIPCS*, the level of expression at 7 dpi was similar in TAG5 and ZG14 for the infected samples. Although the expression level of *EgrIPCS* was lower in ZG14 than in TAG5 at 3 dpi, it was significantly higher at 7 dpi in ZG14. This data provides evidence that ZG14 host may have a delayed defence response compared to TAG5.

## Discussion

### Modulating hormone signaling may result in the moderate resistance observed in TAG5

Whilst a degree of overlap exists between the moderately resistant and susceptible host (Additional file 5: Table S3) with regard to the perception and initial activation of defence, the proliferation of the disease caused by *C. austroafricana* is confined in TAG5. Thus TAG5 may modulate defence responses at various levels that collectively contribute to a degree of resistance.

Investigating the phytohormone responses activated in the moderately resistant TAG5 and susceptible ZG14 suggests a role for various hormones in contributing to defence against *C. austroafricana*. ET and JA are both known to be involved in defence against necrotrophs and a convergence point at which these pathways interlink is through ERF proteins. Whilst the *ERF1* gene is up-regulated in both hosts, the *ERF2* gene is only induced in TAG5. ERF2 is involved in inducing the expression of defence genes that are synchronized by JA and ET such as *PR3* and *PR4* [20,21]. Supporting this, it was found that although both these pathogenicity genes are up-regulated in TAG5 and ZG14, the level of expression was significantly higher in the moderately resistant host and this increased expression could be attributed to *ERF2*. JA is another signaling pathway that has been implicated in necrotrophic defence, however we did not observe an increase of this phytohormone at the metabolite level following infection until 7 dpi. This may be a contributing factor as to why TAG5 is moderately resistant and not fully resistant against *C. austroafricana*.

In a previous study we found that the marker genes of SA, usually associated with defence against biotrophs, were up-regulated in *E. grandis* following challenge with *C. austroafriana* [22]. In accordance, this study further elucidates the role of SA through the potential involvement of this hormone in SAR. In the moderately resistant TAG5, basal levels of SA were found be significantly higher than the susceptible ZG14 basal levels at all the investigate time points. It is possible that higher SA levels may create an SAR response in TAG5 earlier than in the susceptible ZG14. The transcript levels of SAR markers, *AtMES1* and *PNP-A* were up-regulated in the moderately resistant TAG5 at 3 dpi thus supporting this notion. The gene encoding for *PNP-A* was found to be down-regulated in the susceptible ZG14. Plant natriuretic peptides are a group of molecules that are involved in SAR signaling [23]. Although SAR originates through SA, this hormone is not the signal that is transduced through the rest of the host. Instead, methyl salicylate (MeSA) was found to be the systemic signal [24,25]. MeSA is biologically inactive and is converted back to SA in distal tissue to induce SAR via methyl esterase (*AtMES*) [26,27]. The SAR response may be more effective in confining disease spread in the moderately resistant TAG5 than in the susceptible ZG14 due to the higher SA levels.

TAG5 displayed a decrease in GA hormone levels following inoculation whilst the levels remained unchanged in ZG14 at 3 dpi. Gibberelins are a group of hormones that is usually implicated in plant development but have been associated with plant defence. Increased GA levels are associated with enhanced susceptibility towards necrotrophs [28]. GA is under the control of DELLA proteins, part of the GRAS family proteins that inhibit this signaling pathway [19,29]. A closely related transcription factor family, designated as Scarecrow-like (SCL), have been shown to have high similarity to other GRAS family proteins by containing the conserved VHIID and SAW motif [30,31]. In this study we found *SCL13* to be up-regulated in the susceptible ZG14 and suppressed in the moderately resistant TAG5. *SCL3* was found to modulate DELLA expression through repression thus promoting GA biosynthesis [32], and due to the high similarity with *SCL13,* one can postulate that SCL13 may be suppressed in TAG5 to relieve its repression on the DELLA proteins. The combinatorial effects of an increase in *GA2ox* and a decrease of *SCL13* in TAG5 may reduce the levels of GA in this host. Concurrent with this, the up-regulation of *SCL13* may contribute to the suppression of RGA and RGL2 in ZG14.

It is intriguing that from our study both SA and GA levels appear to be implicated in resistance as they are both down-regulated in TAG5 at 3 dpi following pathogen challenge. One possible explanation is the cross-talk between these hormone pathways. DELLA proteins have also been shown to repress SA signaling [28] and thus the possible relief in DELLA repression through *SCL13* could influence the down-regulation of the SA signaling pathway. This potential increase in DELLA’s through the down-regulation of *SCL13* in the moderately resistant TAG5, coupled with an increase in the expression of *WRKY33*, may stimulate the suppression of SA observed at the metabolite level in TAG5.

The hormone responses could also be pathogen induced. It has been shown that pathogens can produce gibberellins as a virulence factor facilitating its own growth and proliferation [33,34]. A preliminary investigation into the genome of *C. austroafricana* suggests that this fungus has the ability to produce gibberellins which may manipulate the GA pathway within the host (Mangwanda *et al.*, Unpublished). GA was first isolated from the pathogenic fungus, *Gibberella fujikuroi* and mutants that were deficient in this hormone production displayed no alteration in development. Thus it has been proposed that pathogens may produce hormones to modulate the defence response of the host. The production of GA could be employed by pathogens to degrade DELLA proteins therefore allowing for successful colonization [11,35]. This maybe a possible scenario occurring in the susceptible genotype.

### Susceptibility could be due to a delay in the expression of defence genes

The ability to detect the pathogen early in defence may afford the host an advantage to activate the correct down-stream response to limit the spread of the pathogen. Susceptibility could either result from the host lacking the required defence genes i.e. uncertainty in activating the correct response or it could be due to a later activation of the defence genes compared to a resistant host [36-39]. It is also plausible that both these scenarios could occur simultaneously. The pattern of a possible delayed defence response was observed in ZG14 compared to TAG5.

*EgrACO1* and *EgrIPCS2* were confirmed to be DE in moderately resistant TAG5 but not in the susceptible ZG14 at 3 dpi. However at 7 dpi the expression level of these candidates in ZG14 is comparable to that of TAG5. *IPCS2* is involved in limiting the spread of PCD which would be beneficial for defence against a necrotrophic pathogen [40-42]. Even in scenarios whereby the expression in TAG5 was higher compared to ZG14 at 3 dpi, as with *EgrOSM34, EgrPR1B* and *EgrPR3,* the expression level was similar for both hosts at 7 dpi. Thereby this indicates that ZG14 may have the required artillery to activate an effective defence response, however the timing has a crucial role. It is evident from the selected genes profiled in this study that TAG5 may be able to activate a quicker and more robust defence response compared to that of ZG14 which may contribute to resistance.

## Conclusion

In summary we show that cell wall modifications, fluctuations in the redox state and the combinatorial effects of phytohormones may contribute to moderately resistant phenotype of *E. grandis* to *C. austroafricana*. Taken together, the patterns that emerge from the transcriptome profiling suggest that the moderately resistant TAG5 is able to regulate its defence responses at various phases. Future work would include further investigation into the *C. austroafricana* genome to explore the pathogenicity mechanism employed by this pathogen. Additionally the data generated in this study illustrates that an integration and coordination of different responses is key in eliciting an effective response in *E. grandis* and extends our understanding of plant defence in woody species.

## Methods

### *E. grandis – C. austroafricana* inoculation trial

*E. grandis* ramets of clones TAG5 and ZG14 (Mondi, South Africa) with an approximate stem diameter of 1 cm (±0.2 cm) were challenged with the fungal pathogen *C. austroafricana* (isolate CMW2113) as described by Naidoo et al. [22]. Briefly, a 5 mm cork borer was used to create a wound on the stem by removing the bark and vascular cambium. The fungus was grown on 2% Malt extract agar (MEA) for 5 days at 28°C and an agar plug corresponding to the size of the wound was placed on the opening. Control plants were mock inoculated with sterile 2% MEA agar plugs. The inoculation site was sealed with Parafilm®. Plants were arranged in a randomized block design and kept in a controlled environment (28°C) for 6 weeks during which lesion lengths were recorded at 24 hpi, 48 hpi, 3 dpi and at 7 dpi. Stem material harboring the lesion as well a small section of surrounding healthy tissue was harvested at 24 hpi, 48 hpi, 3 dpi and at 7 dpi. Three biological replicates consisting of three ramets each were harvested for inoculated and mock-inoculated plants of each clone at the two time points.

### Hormone measurements

SA, JA and GA hormone measurements were performed at 24 hpi, 48 hpi, 3 dpi and 7 dpi on the harvested stem material by the Food & Drug Assurance Laboratories (Pretoria, South Africa) using a modified protocol from [43] with Agela Cleanert C8/SCX cartridges. A total of 0.2 g plant material was accurately weighed into a 50 mL polypropylene tubes and suspended in 2 mL Bieleski’s solvent (75% MeOH : 20% H_2_O : 5% Formic acid). These samples were sonicated (Integral Systems, 50 Hz) for 5 min, and then shook on a shaking platform for another 30 min at RT. The samples were centrifuged at 9500 g for 10 min at 4°C (Beckman Coulter Allegra X-22R centrifuge). The supernatants (SN) were kept and the pellet again re-suspended in 2 mL Bieleski’s solvent, shook and centrifuged. The SN were pooled. For JA and SA analyses, 1 mL of the pooled sample was filtered using nylon Clarinert syringe filters (0.22 μm, 13 mm, Agela Technologies) and placed into a vial. For GA analyses the samples were dried at 60°C in a TurboVap LV (Biotage) concentration evaporator using air. The dried extracts were re-suspended in 1 mL of 10% MeOH containing 0.1% formic acid. Solid phase extraction (SPE) cartridges (C8/SCX, 500 mg, Agela Technologies) were conditioned with 5 mL methanol (MeOH) and then 5 mL H_2_O containing 1 M formic acid. After loading with the samples, the columns were washed with 5 mL H_2_O containing 1 M formic acid. Samples were eluted using 3 mL 100% MeOH. The samples were dried in the concentration evaporator at 60°C, re-suspended with 200 μL 10% MeOH and filtered nylon Clarinert syringe filters (0.22 μm, 13 mm, Agela Technologies) into a vial for UPLC injection. 20 μL injections were made on the autosampler. An ABSCIEX API4000 mass spectrometer with turbo spray source was operated in negative ionization mode using a Shimadzu LC-20 AD UPLC binary pump, SIL20ACXR autosampler, and Shimadzu CTO-10A column oven. The Kruskal-Wallis test (p < 0.05) was applied to test the significance between samples.

### Sample preparation and transcriptome sequencing

Total RNA was extracted from the harvested material using a modified cetyl-trimethyl-ammonium bromide (CTAB) protocol [44]. A schematic of the amount of sample harvested and the process is represented in Figure 3a. Samples were treated with RNase-free DNaseI enzyme (Qiagen Inc, Valencia, CA) and purified using the RNeasy® MinElute Kit (Qiagen Inc, Valencia, CA) according to the manufacturers’ protocol. The concentration and quality of the RNA samples were determined by the Bio-Rad Experion analyzer (Bio-Rad, Hercules, CA). Purified samples of TAG5 and ZG14 (control and inoculated) at 3 dpi were sent to the Beijing Genome Institute (BGI) for RNA-Sequencing using the Illumina Genome Analyser with a 50 bp paired end module (Illumina, San Diego, CA).

### Bioinformatic analyses of transcriptome data

#### Read mapping and transcript quantification

RNA reads obtained from BGI for the moderately resistant TAG5 and susceptible ZG14 were processed through the Galaxy platform [45-47]. The quality of the reads was assessed using FASTQC and FASTQ groomer. Mapping of the reads was performed using Bowtie 2 which aligns the reads, and Tophat v2.0.4 which maps novel splice junctions against the *E. grandis* genome [version 1.1] with the allowance of 2 bp mismatch per 50 bp read and a maximum intron length of 10000 bp. Following mapping, Cufflinks v1.03 was used to quantify the transcript abundance.

#### Differential gene expression and gene ontology enrichment

The identification of significantly DE gene models was performed using Cuffdiff v1.0.3 (FPKM >1000). The lists of DE genes for the moderately resistant TAG5 and susceptible ZG14 were assigned an *Arabidopsis* TAIR 10 annotation based on reciprocal BLAST search and divided into up- and down-regulated datasets for each genotype. These subsets were then evaluated for significant (Benjamini & Hochberg False Discovery Rate (FDR) correction analysis, p < 0.05) over-representation of gene ontology terms using the **Bi**ological **N**etworks **G**ene **O**ntology tool (BiNGO) v2.44 which is a plugin for Cytoscape v2.8.2. To aid in the visualization of the various genes in the context of different metabolic processes, MAPMAN v3.5.1R2 [48] was employed.

### RT-qPCR validation

Total RNA extracted from 3 dpi for TAG5 and ZG14 (control and inoculated) was used for validation of the RNA-sequencing data using real-time quantitative PCR (RT-qPCR). Additionally, total RNA extracted from material harvested at 7 dpi was also investigated for expression patterns of selected genes. First strand cDNA synthesis was performed using the Improm II reverse transcriptase enzyme (Promega, Wisconcin, USA) according to the manufacturers’ protocol. Quantitative PCR was performed as outlined in Naidoo et al. [22] with the exception of different target and reference genes.

The amplification efficiency of each primer pair was determined using a serial dilution set made from a pool of cDNA samples. Relative expression and normalization was conducted using *qBASE*plus v1.0 [49]. Normalization of the target genes was based on the stable expression of the following reference genes: ATP Binding protein (*EgrABP*, Eucgr.I01239) and Zinc ion Binding Protein (*EgrZIB*, Eucgr.D02582). Reference genes were regarded as stable if the mean coefficient of variation (CV) and stability (M) values were below 0.25 and 0.5 respectively. Primers for all genes were designed using Primer Designer 4 v4.20 (Sci Ed Central, Cary, North Carolina, USA) and synthesised by Whitehead Scientific (Cape Town, Western Cape, South Africa). The Shapiro-Wilk’s test was used to test for normality of the data with the statistical software package Analyse-it® (Analyse-it Software, Ltd., Leeds, UK). The Kruskal-Wallis test (p < 0.05) was applied to test the significance between samples.

### Availability of supporting data

The data sets supporting the results of this article are available in the Gene Expression Omnibus repository (GSE67554; http://www.ncbi.nlm.nih.gov/geo/query/acc.cgi?acc=GSE67554).

## Additional files

Additional file 1: Table S1. Summary of statistics obtained for transcriptome profiling of TAG5 and ZG14 challenged with *C. austroafricana*. Additional file 2: Table S2. Summary of significantly differentially expressed genes and their annotations identified from *Eucalyptus grandis* TAG5 and ZG14. Additional file 3: Figure S1. Molecular function GO terms that are over-represented in TAG5 and ZG14. a – GO terms within the up-regulated dataset. b – GO terms within the down-regulated dataset (all terms for this dataset are shown). The y-axis represents the –log_2_(q-value) and the x-axis represents the GO terms within the datasets. Light and dark grey bars are ZG14 and TAG5 respectively. Additional file 4: Figure S2. Cellular component GO terms that are over-represented in TAG5 and ZG14. a – GO terms within the up-regulated dataset. b – GO terms within the down-regulated dataset. The y-axis represents the –log_2_(q-value) and the x-axis represents the GO terms within the datasets. Light and dark grey bars are ZG14 and TAG5 respectively. Additional file 5: Table S3. List of differentially expressed genes that are common between the susceptible (ZG14) and moderately resistant (TAG5) host.

## Abbreviations

ACO: 1-aminocyclopropane-1-carboxylate oxidase
JA: Jasmonic acid
AtMES: Methyl esterase
MEA: Malt extract agar
BP: Biological processes
MF: Molecular function
CC: Cellular component
OSM34: Osmotin 34
DE: Differentially expressed
PNP-A: Plant natriuretric acid
dpi: Days post inoculation
PCD: Programmed cell death
ET: Ethylene
PR1: Basic pathogenesis related 1
EBF: EIN3 Binding F-BOX
PR3: Pathogenesis related protein 3
GA: Gibberellic acid
ROS: Reactive oxygen species
GA2ox: GA 2-oxidases
SA: Salicylic acid
GPX2: Glutathione peroxidase 2
SAR: Systemic acquired resistance
hpi: Hours post inoculation
SCL13: Scarecrow-like 13
IPCS2: Inositol phosphorylceramide synthase 2
wpi: Weeks post inoculation
