# Supplementary material for: Transcriptome and hormone profiling reveals Eucalyptus grandis defence responses against Chrysoporthe austroafricana
Source: BMC Genomics. 2015 Apr 18;16(1):319. doi: 10.1186/s12864-015-1529-x (PMC4405875; doi:10.1186/s12864-015-1529-x)
Supplement: Additional file 1: Table S1. — Summary of statistics obtained for transcriptome profiling of TAG5 and ZG14 challenged with C. austroafricana. [file 12864_2015_1529_MOESM1_ESM.pdf]

Samples	RIN	Total PE Reads	% Mapped reads to *E. grandis* genome			% Mapped reads to *C. austroafrianca* genome	Q20 Percentage (%)	GC Percentage (%)	Expressed genes	Expressed transcripts
			Paired	Singletons	Total					
TAG5 Control BR1	9.6	39,273,762	64.0	12.6	76.6	0.5	97.6	49.06	28448	32308
TAG5 Control BR2	9.7	39,195,029	64.5	13.2	77.6	0.5	97.59	49.2	28122	31914
TAG5 Control BR3	9.5	39,370,778	64.4	13.3	77.7	0.5	97.59	48.93	28239	32083
TAG5 Infected BR1	9.6	37,617,103	63.5	12.0	75.5	2.1	97.75	48.67	28487	32384
TAG5 Infected BR2	9.5	37,390,551	64.2	12.7	76.9	3.2	97.58	49.31	28688	32581
TAG5 Infected BR3	9.5	38,684,116	65.4	13.2	78.6	1.9	97.66	49.41	29829	32152
ZG14 Control BR1	9.6	37,594,917	65.3	13.1	78.4	0.5	97.68	49.02	27823	31644
ZG14 Control BR2	9.7	38,697,190	64.5	12.9	77.3	0.5	97.68	49.06	28043	31917
ZG14 Control BR3	9.6	38,856,446	63.9	12.6	76.5	0.5	98.16	48.76	28053	32003
ZG14 Infected BR1	9.7	38,062,937	63.6	11.5	75	2.9	98.13	49.33	27714	31525
ZG14 Infected BR2	9.6	34,373,634	64.5	11.7	76.2	3.2	98.18	48.36	27889	31707
ZG14 Infected BR3	9.7	36,626,103	64.3	11.5	75.8	3.2	98.13	48.77	27729	31568
