# Supplementary figures and images for: Transcriptome and hormone profiling reveals Eucalyptus grandis defence responses against Chrysoporthe austroafricana
Source: BMC Genomics. 2015 Apr 18;16(1):319. doi: 10.1186/s12864-015-1529-x (PMC4405875; doi:10.1186/s12864-015-1529-x)

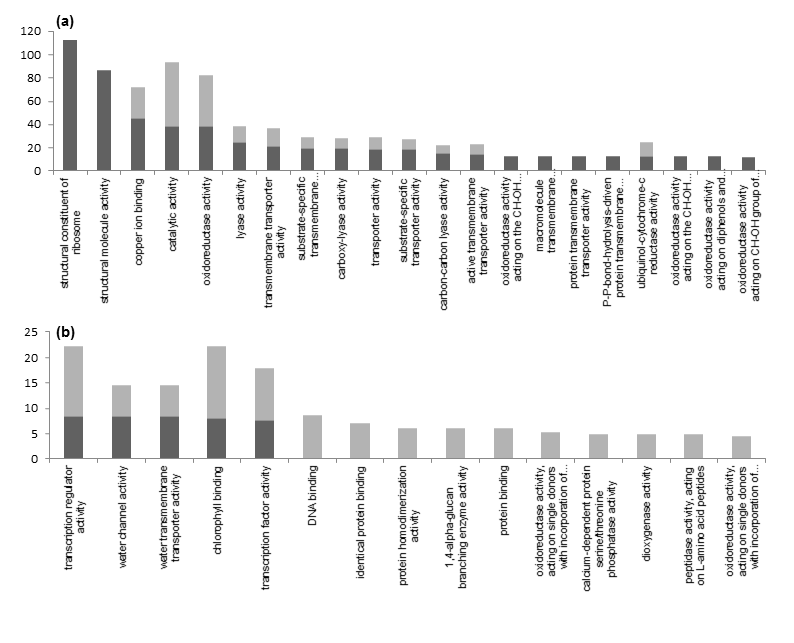

Supplement: Additional file 3: Figure S1. — Molecular function GO terms that are over-represented in TAG5 and ZG14. a – GO terms within the up-regulated dataset. b – GO terms within the down-regulated dataset (all terms for this dataset are shown). The y-axis represents the –log2(q-value) and the x-axis represents the GO terms within the datasets. Light and dark grey bars are ZG14 and TAG5 respectively. [file 12864_2015_1529_MOESM3_ESM.tiff]

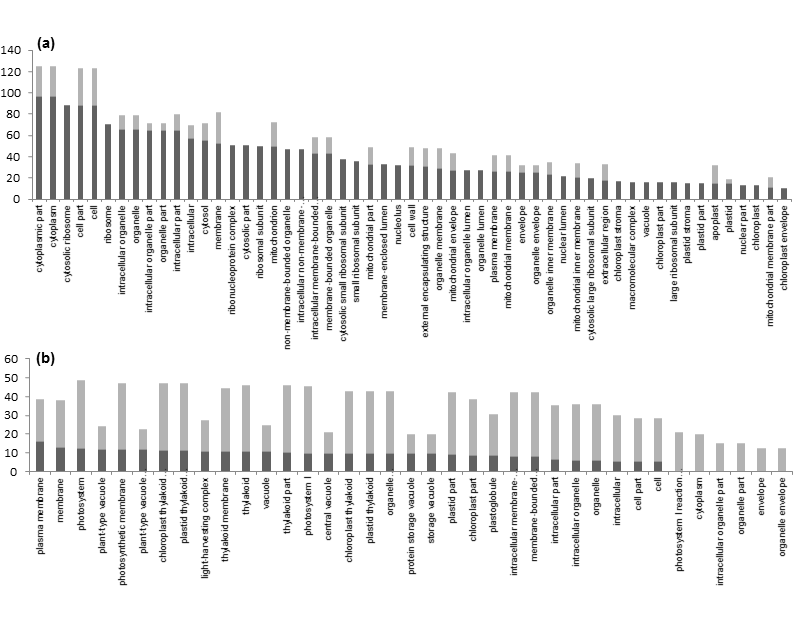

Supplement: Additional file 4: Figure S2. — Cellular component GO terms that are over-represented in TAG5 and ZG14. a – GO terms within the up-regulated dataset. b – GO terms within the down-regulated dataset. The y-axis represents the –log2(q-value) and the x-axis represents the GO terms within the datasets. Light and dark grey bars are ZG14 and TAG5 respectively. [file 12864_2015_1529_MOESM4_ESM.tiff]
